# Biomechanical Determinants of Plaque Erosion: Translational Implications and Precision Care

**DOI:** 10.1016/j.jacbts.2026.101621

**Published:** 2026-07-10

**Authors:** Jason Sangha, Eirinaios Tsiartas, Yuan Huang, Sophie Gu, Kevin Mohee, Fan Zhang, Michael Roberts, Martin Bennett

**Affiliations:** a Department of Medicine, University of Cambridge, Cambridge, United Kingdom; b Department of Applied Mathematics and Theoretical Physics, University of Cambridge, Cambridge, United Kingdom

**Keywords:** biomechanics, computational fluid dynamics, finite element analysis, fluid-structure interaction, plaque erosion, plaque structural stress

## Abstract

Plaque erosion is a major cause of acute coronary syndromes and is characterized by endothelial denudation, fibrous-cap integrity, and platelet-rich thrombosis. Advances in intracoronary optical coherence tomography have enabled in vivo identification of erosion, revealing unique clinical profiles and therapeutic opportunities, including selectively avoiding stents. Increasing evidence implicates local mechanical forces as central drivers of endothelial injury in erosion. Computational biomechanics, including fluid dynamics and emerging optical coherence tomography–based finite element analysis, allow detailed quantification of hemodynamic and structural stresses at plaque surfaces. These models provide mechanistic insights into how flow disturbance, plaque geometry, and wall stress may converge to trigger erosion. Integrating biomechanical signatures with erosion-associated biological pathways may refine risk stratification, guide conservative management, and identify new therapeutic targets. This review integrates fluid and emerging solid mechanics approaches with endothelial biology to provide a comprehensive mechanistic overview of plaque erosion and outlines priorities for translating biomechanics into precision care.

## Highlights

• Plaque erosion is a cause of ACS, with implications for personalized, morphology-based treatment.
• We review the interplay of biological, hemodynamic, and mechanical forces in plaque erosion.
• We describe how biomechanics can identify erosion-prone regions and guide management.
• Validation requires inclusion of biomechanical risk signatures into prospective studies.

**Figure 1:**
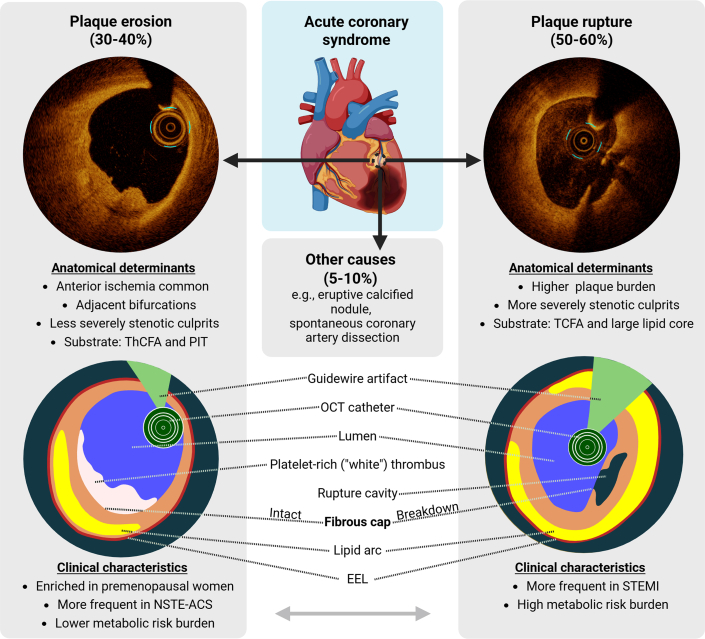
Morphological Mechanisms of Acute Coronary Syndrome Representative OCT images and schematic cross-sections illustrating the key features of plaque erosion (left) and plaque rupture (right), the two dominant substrates of ACS. Created in BioRender, BI (2026), https://BioRender.com/8mrgwnl. EEL = external elastic lamina; NSTE-ACS = non–ST-segment elevation acute coronary syndrome; OCT = optical coherence tomography; PIT = pathologic intimal thickening; STEMI = ST-segment elevation myocardial infarction; TCFA = thin-cap fibroatheroma; ThCFA = thick-cap fibroatheroma.

Plaque erosion has emerged as a major and distinct mechanism of acute coronary syndromes (ACS), challenging the long-standing paradigm that plaque rupture is the predominant cause of coronary thrombosis.^1^, ^2^, ^3^ Initially described in early histopathology studies as a frequent cause of sudden cardiac death, plaque erosion is characterized by thrombus formation on an intact fibrous cap without continuity with the necrotic core (Figure 1).^4^^,^^5^ Intracoronary optical coherence tomography (OCT) has enabled detailed in vivo visualization of culprit plaques, revealing erosion in approximately 30% of ST-segment elevation myocardial infarction cases and nearly half of non–ST-segment elevation myocardial infarction presentations.^1^^,^^2^ The anatomical substrate for erosion appears distinct from plaque rupture, with eroded plaques showing larger lumen sizes, and the majority of lesions comprise thick cap fibroatheroma or pathological intimal thickening, whereas plaque rupture is associated with thin cap fibroatheroma and larger lipid cores.^3^ Consequently, unlike rupture, erosion does not arise from a single, high-risk morphology, and erosion-prone lesions cannot be reliably identified by conventional invasive imaging. This highlights the need for adjunctive plaque assessment and risk prediction tools, with computational modeling of coronary biomechanics garnering interest as a potential means for augmenting the identification of destabilization-prone plaques.^6^

Patients experiencing plaque erosion display distinct demographic and clinical profiles compared with those experiencing rupture, which may provide clues to pathogenesis and treatment. Patients who have erosion exhibit fewer metabolic risk factors such as diabetes, dyslipidemia, and chronic kidney disease,^1^^,^^7^ and although early histopathological studies suggested a female predominance, contemporary intracoronary imaging data show a similar overall sex distribution, with erosion being more frequent only among younger women.^4^^,^^8^ Histologically, erosion is characterized by endothelial denudation overlying a smooth muscle–rich and proteoglycan-rich plaque with an intact fibrous cap, typically accompanied by a platelet-rich rather than fibrin-rich thrombus.^4^^,^^9^^,^^10^ This pathology differs fundamentally from plaque rupture, which results from structural failure of an inflamed fibrous cap overlying a necrotic core.^4^^,^^11^ The associated inflammatory sequelae also differ, with rupture typically characterized by macrophage- and lymphocyte-mediated inflammation, whereas erosion displays a relatively mild inflammatory response dominated by neutrophil infiltration.^5^^,^^11^^,^^12^

The central role of endothelial injury in erosion pathophysiology increasingly implicates local mechanical and hemodynamic forces as key drivers, and understanding the interplay between plaque morphology and flow disturbance is possible through computational biomechanics.^13^ Modeling approaches such as computational fluid dynamics (CFD), finite element analysis (FEA), and fluid-structure interaction (FSI) permit detailed quantification of biomechanical parameters at the plaque level,^13^^,^^14^ but despite these insights, current ACS guidelines make no explicit reference to plaque erosion, highlighting a persistent translational gap.^15^^,^^16^ In this review we discuss how biomechanical determinants of plaque erosion, including emerging solid mechanics approaches, combined with endothelial biology, can clarify the mechanisms underlying erosion and identify translational research directions.

## Fundamentals of Coronary Biomechanics

### Overview of biomechanical forces acting on the arterial wall

Stress and strain are key concepts in understanding how a biological tissue responds to forces. Stress can be considered as the interparticle forces attempting to resist deformation in response to external forces and is normalized to unit area, whereas strain is the resulting deformation or change in length. Blood pressure exerts a perpendicular force on the coronary arterial wall resulting in radial wall stress, and consequently circumferential wall stress, with typical values in the range of 1 to 100 kPa.^17^ When circumferential wall stress is located at a region of an atherosclerotic plaque, it is termed “plaque structural stress” (PSS), and higher values of PSS have been associated with plaque rupture.^18^, ^19^, ^20^ The law of Laplace describes the behavior of hollow thin-walled structures and governs the relationships between circumferential stress, blood pressure, lumen radius, and wall thickness (Central Illustration); consequently, PSS increases with lumen area and decreases with luminal stenosis and increasing plaque thickness.^21^ In addition, blood flow through the vessel exerts frictional stress on the endothelium, termed “endothelial shear stress” (ESS). Shear stress is 10^5^ orders of magnitude less than PSS (typically 1-7 Pa), but given that endothelial cells express mechanosensors, ESS exerts important effects on intracellular signaling pathways and is implicated in both plaque development and processes including vascular remodeling and tone.^22^ In addition to absolute ESS, the endothelial shear stress gradient (ESSG), which describes change in ESS between adjacent areas, is also of importance,^23^ whereas the oscillatory shear index (OSI) describes the change in ESS over a single cardiac cycle and again has been linked to plaque development.^24^

**Central Illustration:**
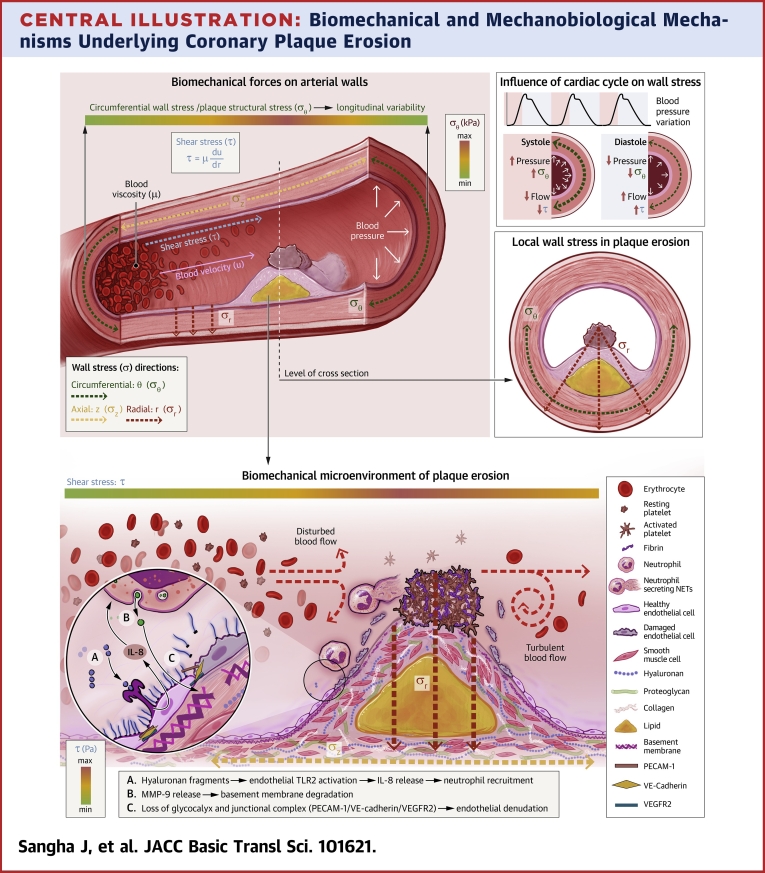
Biomechanical and Mechanobiological Mechanisms Underlying Coronary Plaque Erosion (Top left) Schematic overview of the principal biomechanical forces acting on the coronary arterial wall. Blood viscosity (μ) and velocity (u) generate endothelial shear stress (τ), whereas intraluminal pressure and vessel geometry determine circumferential (plaque structural) wall stress (σ_θ_), together with axial (σ_z_) and radial (σ_r_) wall stresses. (Top right) The cardiac cycle dynamically modulates local biomechanical forces, with systolic increases in pressure resulting in higher circumferential wall stress and diastolic increases in flow augmenting endothelial shear stress. The accompanying cross-sectional view illustrates the distribution of circumferential and radial wall stresses across an eroded plaque. (Bottom) Mechanobiological microenvironment of plaque erosion. Disturbed blood flow over an intact extracellular matrix-rich fibrous cap promotes endothelial dysfunction and denudation, facilitating platelet-rich thrombus formation. Hyaluronan fragmentation activates Toll-like receptor 2 signaling, leading to interleukin-8-mediated neutrophil recruitment, matrix metalloproteinase-9 release, basement membrane degradation, loss of endothelial junctional complexes (PECAM-1, VE-cadherin, and VEGFR2), and neutrophil extracellular trap formation, amplifying thrombo-inflammatory propagation. IL-8 = interleukin-8; MMP-9 = matrix metalloproteinase-9; NET = neutrophil extracellular trap; PECAM-1 = platelet endothelial cell adhesion molecule-1; TLR2 = toll-like receptor-2; VE-cadherin = vascular endothelial cadherin; VEGFR2 = vascular endothelial growth factor receptor-2.

Although ESS and PSS are the most widely investigated biomechanical forces in coronary plaques, other stresses and strains acting on the arterial wall are of note. Radial wall strain can be derived from invasive angiography images by quantifying vessel wall deformation during the cardiac cycle and has recently been shown to predict future cardiovascular events in angiographically mild to intermediate stenotic lesions.^25^

## Biomechanical Modeling and Clinical Perspectives

### Fluid mechanics

**Figure 2:**
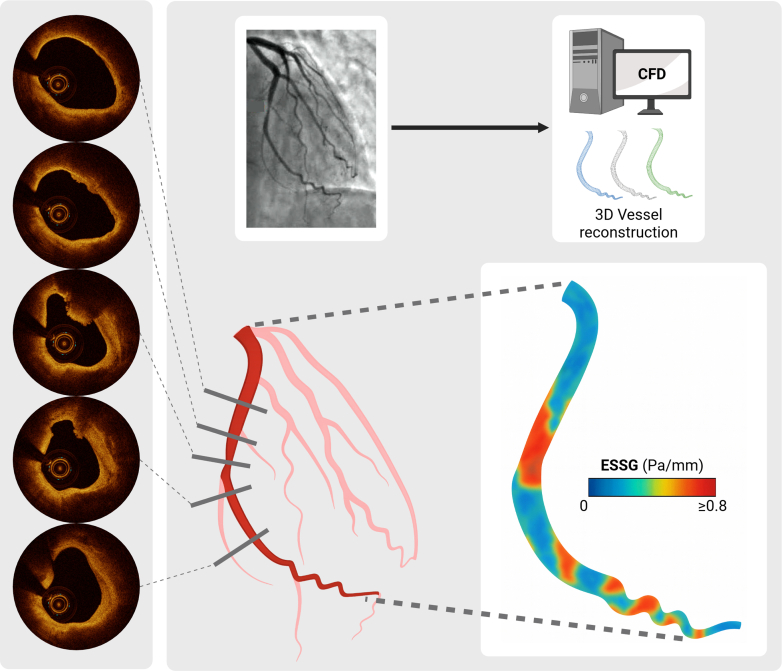
Computational Fluid Dynamics Pipeline and Representative Flow Field at an Erosion Site Intracoronary OCT pullbacks are co-registered with angiographic projections, and a 3D reconstruction of the coronary vessel generated. The reconstructed lumen geometry is then processed for CFD analysis to derive local hemodynamic indices. The example shown illustrates the distribution of ESSG, with regions of elevated ESSG (red) marking areas of spatially heterogeneous flow associated with plaque erosion. Created in BioRender. B, I. (2026) https://BioRender.com/r8brp13. 3D = 3-dimensional; CFD = computational fluid dynamics; ESSG = endothelial shear stress gradient; Pa = Pascal.

The observation that coronary plaques cluster preferentially at ostia, bifurcations, and inner curvatures—despite the systemic effects of cardiovascular risk factors—prompted the application of CFD-based approaches to the coronary tree to quantify changes in hemodynamic parameters along the artery. Calculating indices such as ESS requires 3-dimensional lumen reconstruction from multimodal imaging—a combination of either computed tomography or biplane invasive angiography together with intracoronary imaging (Figure 2). The reconstructions are then filled with grid points which represent blood, as part of computational meshing. Luminal shear stress is then calculated by solving Navier-Stokes equations, which govern the motion of arteries and blood flow within defined boundary conditions.

The majority of intracoronary CFD studies to date have focused on shear stress and its relation to plaque progression and destabilization, and—in contrast to solid mechanics studies—reported associations have been contradictory. Low ESS has been associated with plaque development, progression, and major adverse cardiovascular events (MACE),^26^ whereas high ESS and high ESSG are also associated with MACE.^23^ Currently, although there are commercial and open-source software platforms that can simulate intracoronary shear stress (eg, Ansys Fluent, OpenFOAM), ESS and related indices cannot be applied in real time in the cardiac catheterization laboratory because of long computational processing times.

### Solid mechanics

**Figure 3:**
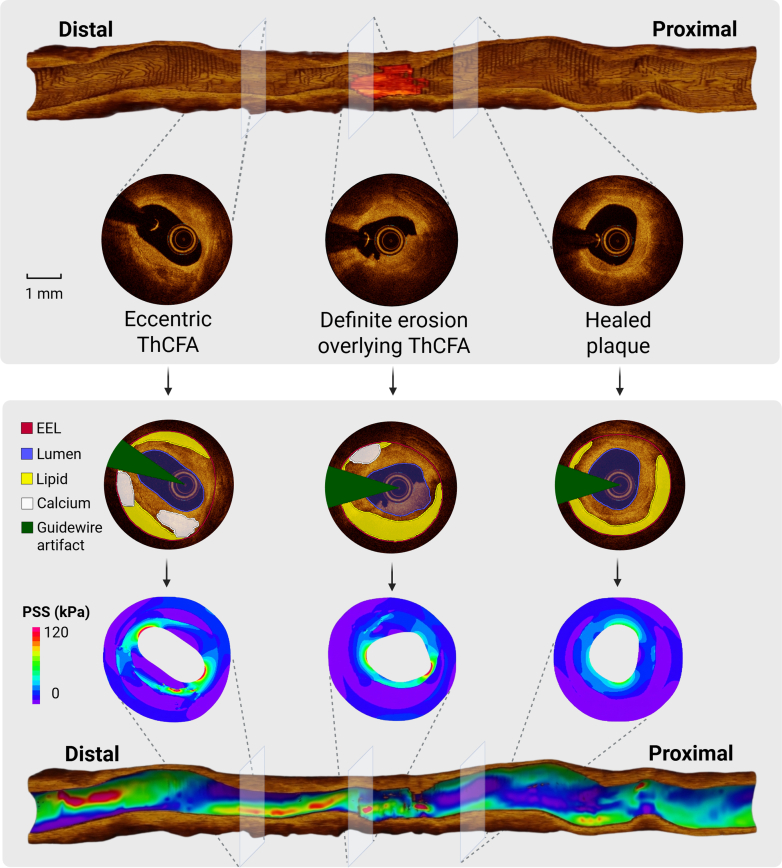
Finite Element Analysis Workflow and PSS Map Derived from an OCT Pullback Demonstrating Plaque Erosion (Top panel) 3D reconstruction of OCT pullback, showing definite plaque erosion overlying a ThCFA, with healed plaque upstream, and an eccentric ThCFA distally. (Lower panel) Artificial intelligence-based segmentation of plaque components (top row), after which finite element analysis simulates PSS under dynamic blood pressure loading conditions. Band plots are generated to visualize areas of high PSS (middle row) shown in red. PSS is low upstream of the erosion in the healed plaque region, and areas of high PSS are located at and distal to the erosion site, as visualized in the pseudo-3D reconstruction. Created in BioRender. B, I. (2026) https://BioRender.com/mewgd3k. EEL = external elastic lamina; kPa = kilopascal; PSS = plaque structural stress; ThCFA = thick-cap fibroatheroma.

Circumferential wall stress (ie, PSS) is the most investigated solid mechanics domain metric in coronary arteries and can be calculated using the engineering technique of FEA. Multiple variables such as plaque geometry and composition—derived from intracoronary imaging—and blood pressure are synthesized to calculate stress (Figure 3). Tissue material properties are assigned to plaque histologic elements (eg, fibrous tissue, lipid, calcium) based on ex vivo tensile testing of coronary artery samples.^27^^,^^28^ Plaque components are segmented frame-by-frame, and FEA simulation can then be performed to estimate PSS. Invasive intracoronary imaging—particularly virtual histology-radiofrequency intravascular ultrasound (VH-IVUS) and optical coherence tomography (OCT)—allows the delineation of plaque components and geometry in vivo. These modalities have provided the basis for in vivo FEA modeling, whereas FSI modeling combines both solid and fluid mechanics.^29^

To date, clinical studies of PSS have focused almost exclusively on plaque rupture. For example, high PSS derived from VH-IVUS imaging predicted clinical presentation (troponin positive ACS vs stable angina) in high-risk plaques.^30^ Subsequently, PSS was found to be higher in ruptured vs nonruptured fibroatheromas delineated by OCT^20^^,^^31^ and in high-risk morphology lesions (plaque burden >70% and MLA <4 mm^2^) which are responsible for MACE.^18^^,^^19^ Although PSS can be determined by plaque tissues, geometric features of the lumen-plaque interface can also increase PSS. For example, eccentric plaques and lumen curvature increase PSS and are associated with cardiac events.^32^

Although these techniques provide estimated plaque stress, future real-time clinical application of PSS in the cardiac catheterization laboratory will depend on rapid, accurate, automatic frame-by-frame OCT image segmentation. As a single coronary lesion can measure hundreds of frames, manual tissue component segmentation is not practical, and automated coupling to engineering software is challenging. We have therefore developed “AutoOCT,” an artificial intelligence–based deep learning algorithm for real-time OCT image segmentation.^33^ Although manual input is currently required to correct segmentations around thrombus, coupling of AutoOCT to FEA yields a near-real-time workflow to investigate erosion (Figure 3).

## Bridging Biomechanics and Erosion Pathophysiology

### From biological observations to mechanical hypotheses: the maladaptive endothelial response

Plaque erosion has been established as a distinct pathophysiological process, represented by endothelial denudation and platelet aggregation over an otherwise intact fibrous cap with rich extracellular matrix (ECM).^4^^,^^9^, ^10^, ^11^ However, whether the loss of endothelial cell markers represents loss of cells - or specific endothelial proteins in intact cells - is unclear, raising a central mechanistic question: *What precipitates endothelial loss in the absence of cap rupture?* Increasing evidence points to mechanical and hemodynamic forces as key triggers that convert a stable anticoagulant endothelium into a thrombogenic surface.^1^^,^^23^^,^^34^

The endothelium is a dynamic mechanosensory layer, translating shear and tensile forces into biochemical responses. This process is mediated by complexes formed by platelet endothelial cell adhesion molecule-1 (PECAM-1), vascular endothelial (VE)-cadherin, vascular endothelial growth factor receptor 2 (VEGFR2), integrins, and the glycocalyx, which relay mechanical cues into downstream signaling pathways.^35^ Under physiological laminar flow, such signaling maintains vascular homeostasis by promoting nitric oxide release, suppressing inflammation, and aligning endothelial cells with flow direction.^35^

When ESS deviates from physiological ranges, these same pathways become maladaptive, leading to cytoskeletal deformation, junctional disruption, and ultimately endothelial apoptosis or detachment, a microscopic hallmark of erosion.^35^, ^36^, ^37^, ^38^ Although these mechanisms have been characterized in experimental models of endothelial dysfunction and atherogenesis, they have not yet been demonstrated directly in human plaque erosion. Current concepts therefore draw on broader vascular mechanobiology, forming a cohesive framework in which a range of abnormal mechanical forces initiate endothelial injury and predispose to erosion (Central Illustration).

### Flow disturbance and mechanoinflammatory activation

Eroded plaques exhibit an inflammatory profile characterized by functional activation of innate immune pathways within the culprit site.^39^, ^40^, ^41^ For example, local and systemic absolute neutrophil counts are similar in erosion and rupture, but neutrophils demonstrate upregulated toll-like receptor-2 (TLR2) expression in erosion, and biologically relevant downstream consequences of TLR2 activation, including matrix metalloproteinase-9 (MMP9) release, are confined to the local culprit plaque microenvironment. In parallel, thrombus aspirates overlying eroded plaques show local enrichment of hyaluronidase-2 (HYAL2) and its substrate hyaluronan, a possible source of endogenous TLR2 ligands at the blood-thrombus interface.^39^

Local hemodynamics provide a context for this compartmentalized activation. Erosion is frequently linked to bifurcation and geometrically complex coronary regions where shear stress is spatially heterogeneous,^37^^,^^42^ and mechanotransduction studies show that disturbed or oscillatory shear promotes a proinflammatory endothelial phenotype compared with stable laminar flow.^35^ As shown in vitro, ECM fragmentation in this setting could generate hyaluronan components that can act as endogenous TLR2 ligands, whereas protease activity including MMP9 could contribute to basement membrane degradation and endothelial detachment.^9^^,^^36^^,^^39^^,^^40^ Bifurcation-associated disturbed flow has also been associated with local CD8^+^ T-cell-mediated immune activation in plaque erosion, linking local hemodynamics to adaptive immune activation.^42^ These complementary human and experimental observations support a cohesive model whereby disturbed coronary flow localizes immune-endothelial interactions, although this has not yet been directly demonstrated in vivo.

In addition to these mechanobiological processes, several other contributors to plaque erosion have been proposed. Coronary vasospasm may induce transient endothelial injury through abrupt alterations in vascular tone and local shear conditions, whereas systemic and local endothelial dysfunction, particularly in the context of smoking, can impair endothelial integrity and promote a prothrombotic surface. Indeed, smoking-related oxidative stress and inflammatory activation have been consistently associated with erosion-prone phenotypes.^43^ These processes are likely to interact with local hemodynamic and structural stresses, lowering the threshold for endothelial denudation and thrombosis. Accordingly, plaque erosion should be considered a multifactorial process in which biomechanical forces act in concert with systemic and local biological factors. However, such dynamic biological events are not captured in most current biomechanical models, which typically rely on static reconstructions and assumptions of steady or simplified flow conditions.

### The biomechanical-thrombotic continuum

Following endothelial dysfunction, the subendothelial matrix provides an adhesive substrate for platelet tethering, especially under the extremes of shear stress gradients.^44^^,^^45^ Activated platelets release proinflammatory mediators and can amplify leukocyte recruitment, creating a self-reinforcing prothrombotic and proinflammatory environment.^46^ A key effector in this process may be neutrophil extracellular traps (NETs), web-like chromatin structures extruded during a specialized form of cell death termed NETosis.^40^^,^^47^ NETs not only entrap platelets and propagate mural thrombus formation but also carry tissue factor, myeloperoxidase, and histones, which can further injure the endothelium and perpetuate thrombosis (Central Illustration).^47^ In experiments designed to mimic human plaque erosion, the combination of a smooth muscle–rich and hyaluronan-rich intima with flow perturbation provoked endothelial loss and thrombus formation, effects mitigated by granulocyte depletion, genetic loss of TLR2, or inhibition of NET formation.^12^^,^^48^ However, while these findings suggest a causal link between NETosis and flow-mediated erosion, they are mainly derived from murine carotid models rather than human coronaries, and proof requires similar findings in human eroded plaques.

### Biomechanics as a potential unifying framework

Together, these findings delineate a mechanobiological continuum in which endothelial injury, immune activation, and thrombosis are dynamically interconnected. Local mechanical forces, shaped by anatomical features and upstream biological factors, may contribute both to the development of erosion-prone plaque phenotypes and to the acute triggering of endothelial denudation, acting in conjunction with a permissive prothrombotic milieu rather than as sufficient triggers for plaque erosion. Within this context, mechanical stresses may initiate endothelial loss, whereas downstream NETosis, complement activation, and possibly endothelial-to-mesenchymal transition amplify thrombus propagation on the exposed matrix. This scenario differs from the rupture phenotype, in which low ESS is more strongly linked to plaque progression, whereas high PSS is implicated in the eventual cap failure. Although attractive, most evidence for this sequence of events derives from reductionist animal models or indirect biomarker studies rather than direct human observation. Similarly, current experimental designs typically isolate individual components such as flow disturbance, TLR2 signaling, or NET formation, without reproducing their integrated sequence, highlighting both the conceptual coherence and translational limitations of the present erosion paradigm.

Understanding this interplay underscores the importance of quantifying not only local hemodynamic forces but also the structural stresses and strain patterns acting on the intact fibrous cap, which may modulate endothelial stability even in the absence of cap rupture. Computational biomechanics offers a means to integrate these processes, linking plaque geometry, shear stress, and wall stress and strain to endothelial dysfunction, and thus provides a critical framework for interpreting and predicting where erosion is most likely to occur. We have therefore systematically reviewed all available studies that have modeled biomechanical determinants of plaque erosion.

## Methods

We systematically reviewed the literature using PubMed and Scopus until September 2025 to identify original human studies using computational modeling to investigate coronary plaque erosion biomechanics, based on predefined eligibility criteria (Supplemental Table 1). The detailed search strategy can be found in Supplemental Table 2. Titles and abstracts were screened by 2 independent reviewers, with full-text assessment and data extraction performed on a previously piloted extraction tool. Conflicts were resolved by consensus.

Given the heterogeneity of study designs, modeling techniques, and reported measures, findings were synthesized narratively and grouped by primary modeling approach (fluid, solid, or combined), with emphasis on recurring mechanistic themes.

## Results

**Figure 4:**
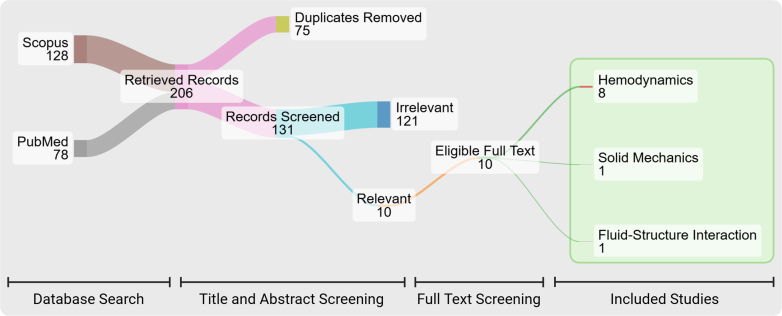
PRISMA Flowchart of Study Selection Process Created in BioRender. B, I. (2026) https://BioRender.com/xhf9nsy.

After deduplication, 131 abstracts were screened, of which 10 met inclusion criteria (Figure 4, Table 1). The majority (n = 8) used CFD to characterize the hemodynamic environment of plaque erosion, 1 used FEA and 1 FSI modeling.

**Table 1:** Characteristics and Summary of Available Studies on Plaque Erosion Biomechanics

First Author (Year)	Imaging Modality	Modeling Approach	No. of Lesions	Control Group	Main Findings
Campbell et al (2013)^49^	OCT (in vivo) and angiography (ex vivo; separate explanted hearts cohort)	CFD	In vivo component: 3 erosions; ex vivo component: 19 (13 erosions, 6 ruptures)	Noncontrolled	Erosions occurred at sites of relatively low ESS (<3 Pa; maximum <12 Pa), with no consistent link to OSI or curvature. Transient helical flow occasionally observed but not unique to erosions. Upstream bifurcations seen in 8/13 autopsy erosion cases.
Campbell et al (2014)^50^	Histology (ex vivo)	FEA	74 (33 erosions, 12 ruptures, 15 TCFA, 14 ThCFA)	Noncontrolled	Stress/strain distribution similar across plaque phenotypes; stress maxima diffuse in erosions. Inflammatory markers (TUNEL, MMPs, CD68) lower in erosions; no strain-inflammation correlation. Erosions more cellular, SMC-rich, less calcified, smaller vessels; more Factor VIII–positive cells.
Yamamoto et al (2019)^38^	OCT (in vivo)	CFD	18 erosions	Noncontrolled	Thrombus formed in the transition between high and low shear zones. In 78%, it originated at peak ESS/ESSG (throat) and extended distally toward high OSI/low ESS. Proximal erosions showed multiple ESS peaks and steeper distal shoulders.
Thondapu et al (2021)^23^	OCT (in vivo)	CFD	37 (18 erosions, 19 ruptures)	Noncontrolled	Erosions linked to STEMI (94%) and smoking (78%). Occurred distally with higher ESS/ESSG and lower OSI vs nonerosion (all *P* < 0.001). Multivariable predictors: high ESS, ESSG, OSI. Compared with rupture: lower ESSG, higher OSI.
McElroy et al (2021)^51^	OCT (in vivo)	CFD	17 erosions	Noncontrolled	In 94% of erosions, thrombus was proximal to or overlying the point of maximal stenosis. Erosion sites showed ≈5× higher mean ESS, 20-22× higher peak ESS, and 5-6× higher ESSG, with 2-4× lower RRT vs reference. OSI unchanged. Three hemodynamic phenotypes identified based on ESS/OSI patterns.
Hakim et al (2023)^34^	OCT (in vivo)	CFD	24 erosions	22 controls matched on MLA and reference luminal area	Erosion plaques had higher maximum ESS and ESSG (any, circumferential, upslope; all *P* < 0.01). Maximum ESS and ESSG independently predicted erosion. Thrombus location aligned with plaque slope (proximal erosion: steeper upslope, distal erosion: steeper downslope). ESSG correlated with slope steepness (*r* ≈ 0.3-0.4). There was no difference in minimum ESS between erosion and stable plaques.
Russo et al (2023)^52^	OCT (in vivo)	CFD	25 (11 erosions, 14 ruptures)	24 chronic coronary syndrome controls; No matching criteria	IFC (erosion) plaques had intermediate ESS (2.5 Pa) and narrower ESS range vs stable or ruptured lesions. IFC showed higher EDN1/LGALS8 and lower ADAMTS13 than RFC. In RFC, ESS correlated with TNFα and MMP9 (positive) and EDN1 (negative); no correlations in IFC.
Ahmed et al (2024)^53^	OCT (in vivo)	CFD	32 erosions	Noncontrolled	Low ESS correlated with greater T-cell infiltration (*r* ≈ −0.4). High ESSG and steeper plaque slope associated with increased CD8^+^/NK cells and proinflammatory cytokines (IL-6, IL-1β, IL-2, MIP-1β). IL-4 inversely related to ESSG/slope.
Zhu et al (2025)^29^	OCT (in vivo)	FSI	8 erosions	Noncontrolled	Combined ESS, lipid %, and thrombus burden best predicted outcomes (AUC: 0.96). ESS alone strongest single predictor (AUC: 0.70). Higher ESS and thrombus burden correlated with adverse events; wall stress/strain differences nonsignificant.
Kim et al (2022)^54^	OCT (in vivo)	CFD	23 erosions	Noncontrolled	ESS and ESSG higher at erosion vs nonerosion sites at all time points (*P* < 0.01) and remained stable over 12 months. OSI unchanged. Thrombus burden decreased during follow-up; stenosis and minimal flow area stable.

AUC = area under the receiver-operating characteristic curve; CD = cluster of differentiation; CFD = computational fluid dynamics; EDN1 = endothelin-1; ESS = endothelial hear stress; ESSG = endothelial shear stress gradient; FEA = finite element analysis; FSI = fluid-structure interaction; IFC = intact fibrous cap; IL = interleukin; LGALS8 = gene encoding Galectin-8; MIP = macrophage inflammatory protein; MLA = minimal lumen area; MMP = matrix metalloproteinase; NK-cell = natural killer-cell; OCT = optical coherence tomography; OSI = oscillatory shear index; RFC = ruptured fibrous cap; RRT = relative residence time; SMC = smooth muscle cell; STEMI = ST-segment elevation myocardial infarction; TCFA = thin-cap fibroatheroma; ThCFA = thick-cap fibroatheroma; TNFα = tumor necrosis factor-alpha; TUNEL = terminal deoxynucleotidyl transferase deoxyuridine triphosphate nick end labeling assay.

### Fluid mechanics and plaque erosion

CFD studies have sought to characterize the local hemodynamic milieu of plaque erosion, using OCT reconstructions to quantify indices such as ESS, ESSG, and OSI.^23^^,^^34^^,^^38^^,^^49^^,^^51^^,^^53^^,^^54^ As mentioned earlier, these hemodynamic parameters modulate endothelial integrity, inflammation, and thrombogenicity. Across studies, plaque erosions arose in regions of elevated and spatially heterogeneous shear stress, although precise proximal or distal localization along the plaque shoulder varied, likely reflecting geometry, stenosis severity, and modeling assumptions.^23^^,^^34^^,^^38^^,^^51^

### Shear stress magnitude and gradients

Building on these patterns, detailed CFD analyses linked specific shear metrics to erosion-prone regions. For example, in a study of 18 patients with ACS due to plaque erosion, ESS and ESSG were significantly higher at the site of thrombus than at the adjacent nonerosion segments, but the opposite was observed for OSI, with significantly lower values at the erosion site.^23^ In multivariable analysis, higher values of ESS, ESSG, and OSI each independently predicted erosion, but, given that high ESS would be expected to be seen together with low OSI, these findings are biologically counterintuitive and may represent a confounding rather than a real mechanism. Another study described 5-to 20-fold elevation in shear magnitude and corresponding 2-to 4-fold reductions in relative residence time, indicating rapid, unidirectional flow and reduced near-wall particle residence at erosions sites.^51^ A matched-control model confirmed these associations and linked ESSG to steeper plaque upslopes at erosion sites.^34^ When assessed longitudinally, elevated shear conditions persisted without significant temporal variation over 12 months of conservative management, suggesting a stable local flow phenotype.^54^ To illustrate these relationships, Figure 2 depicts the CFD workflow and a representative flow field highlighting the spatial heterogeneity of ESSG, with abrupt transitions between low and high values corresponding to the steep shear gradients observed at erosion sites. An earlier study using simplified geometric assumptions of elliptical vessel cross sections and a small (n = 3) in vivo OCT cohort reported erosions under relatively low ESS (<3 Pa) without a clear link to curvature or OSI, likely reflecting methodological limitations.^49^

### Oscillatory shear and flow instability

While the magnitude and gradients of shear stress were consistently linked to erosion-prone regions, the role of oscillatory flow patterns was less uniform. Lower OSI values were reported at erosion sites compared with nonerosion regions, whereas other analyses found no significant OSI differences between erosion and reference segments.^23^^,^^51^^,^^54^ Despite this, a subset of lesions exhibiting paradoxical elevation in both ESS and OSI has been seen, with thrombus extending beyond the point of maximal stenosis, suggesting that flow reversal or recirculation may influence thrombus propagation rather than erosion onset.^51^ Other studies similarly observed that thrombus frequently originated at the high-shear “throat” of the stenosis and extended distally toward regions of high OSI and low ESS, highlighting the downstream influence of disturbed flow,^38^ whereas transient helical flow structures with no consistent OSI association have also been observed.^49^ Collectively, these findings suggest that plaque erosion localization may be more closely associated with regions of elevated ESS and steep gradients, whereas oscillatory or recirculating flow may contribute more to thrombus extension and spatial distribution.

### Spatial distribution of erosions along the plaque

Erosion-site location along the plaque is variable, but studies report a consistent relationship with sharp geometric transitions, rather than a fixed proximal or distal position. For example, some studies reported erosions predominantly at distal shoulders, whereas others found thrombus formation proximal to or over the point of maximal stenosis.^23^^,^^51^ In detailed flow mapping, thrombus was often seen to initiate at the high-shear “throat” and extend distally toward regions of high OSI and low ESS, suggesting dynamic shear transitions along the lesion.^38^ Subsequent analyses linked these positional differences to plaque slope direction, with erosions on steeper upslopes tending to occur proximally and those on steeper downslopes distally.^34^ Although some studies showed no clear curvature or positional preference,^49^ local vessel or plaque morphology associated with steep shear gradients appeared to influence erosion susceptibility.

### Hemodynamic-biological correlations

Two studies integrated hemodynamic modeling with molecular or immunologic profiling, offering mechanistic insights into how shear forces could influence endothelial behavior. Low minimum ESS was linked with greater T-cell infiltration at the culprit site and higher local concentrations of proinflammatory cytokines, whereas high ESSG and steeper plaque slopes correlated with cytotoxic immune activation locally and suppression of systemic anti-inflammatory interleukin-4 expression.^53^ In a transcriptomics study, patients with plaque erosion characterized by intermediate ESS profiles displayed a distinct peripheral blood mononuclear cell gene expression profile, characterized by upregulation of endothelial stress markers (endothelin-1/EDN1, galectin-8/LGALS8) and downregulation of metalloprotease ADAMTS13. The opposite was observed in rupture cases, reflecting a differential stress-response phenotype linked to hemodynamic conditions.^52^ Together, these findings might link the mechanical microenvironment of erosion to endothelial activation and local inflammation, suggesting a continuum between hemodynamic stress and immune modulation at the plaque surface. This proinflammatory environment might facilitate neutrophil recruitment and NETosis, a process implicated in thrombus formation in plaque erosion.^55^, ^56^, ^57^

### Summary of flow hemodynamic insights

Across CFD-based analyses, plaque erosion consistently occurred within regions of elevated and spatially heterogeneous shear stress, typically near abrupt geometric transitions such as the stenotic throat or plaque shoulders (Figure 2). Although oscillatory flow patterns showed variable associations, erosions generally arose within stable, high-shear environments with minimal flow reversal. These hemodynamic conditions could promote endothelial activation, local inflammation, and neutrophil-mediated thrombosis, defining a fluid dynamics sequence in which subsequent solid mechanical stresses could contribute to plaque erosion.

### Solid mechanics and plaque erosion

There are few solid mechanics studies of plaque erosion, in part because current in vivo diagnosis relies on invasive OCT imaging and frame-by-frame manual tissue segmentation, followed by coupling to FEA simulation. A single postmortem histological study found similar relative structural stress and strain patterns between plaque erosion cases and controls.^50^ In contrast, an in vivo OCT-based study found that average PSS was significantly lower in erosion vs control lesions (83.2 vs 107.8 kPa, n = 8).^29^

## Limitations of Biomechanical Modeling

Biomechanical modeling for plaque erosion has several important limitations that relate to the imaging modality, the presence of thrombus, and biomechanical assumptions. Firstly, the ability to assess plaque composition and geometry is constrained by the spatial resolution of intravascular imaging. Intravascular ultrasound provides excellent penetration depth for accurate evaluation of plaque burden, particularly in attenuating plaques; however axial resolution is limited (100-150 μm), and heavy calcification obscures underlying plaque and vessel components. In contrast, although OCT has high resolution, penetration depth is lower, and signal attenuation obscures underlying structures in lipid-rich plaques. Multimodality imaging catheters combining OCT with near-infrared spectroscopy may overcome these challenges^58^ and also demonstrate plaque microcharacteristics such as fibrous cap thickness, but studies using these catheters for biomechanical studies are limited.

To date, studies simulating the mechanical conditions leading to plaque erosion have also ignored the effects of the thrombus. A thrombus can alter the mechanical loads within the eroded plaque, although the relevance of this to detecting a biomechanical signature to identify erosion-prone plaques is unclear. Furthermore, in contrast to plaque rupture—wherein failure of the fibrous cap is temporally tied to high PSS, with adverse shear stress contributing to plaque progression and fibrous cap thinning via mechanotransduction pathways—it is unclear at what stage of plaque development and destabilization biomechanical factors are most influential in erosion.

There are several important modeling assumptions in CFD, FEA, and FSI approaches for studying plaque erosion. Most studies on shear stress assume that blood is a Newtonian fluid with constant viscosity irrespective of velocity and that the arterial wall is rigid with constant nonpulsatile blood flow through the vessel. For PSS, FEA computational models are 2-dimensional, limiting the full representation of complex plaque geometry and 3-dimensional stress distributions, and frames containing side branches are excluded in both CFD and solid mechanics approaches, to avoid violation of modeling assumptions. FSI combines fluid and structural mechanics and can ameliorate the effects of these assumptions, but it is computationally expensive, limiting future real-time applicability.

## Clinical Implications and Future Directions

### Recognizing and managing plaque erosion

The Effective Anti-Thrombotic Therapy Without Stenting: Intravascular Optical Coherence Tomography-Based Management in Plaque Erosion (EROSION) study demonstrated that selected patients with ACS who have OCT-confirmed erosion and <70% residual stenosis can be stabilized with intensive antithrombotic therapy alone, achieving substantial thrombus regression without routine stenting.^59^ Follow-up studies have confirmed the durability of this approach, with >90% of patients remaining event-free at 4 years,^60^ although larger cohort analyses showed that clinical heterogeneity exists within this phenotype, with older age, greater stenotic area, and higher thrombus burden predicting worse long-term outcomes.^61^ Comparative OCT studies indicate that erosion is also associated with delayed vascular healing after drug-eluting stent implantation, characterized by thinner neointima, higher proportions of uncovered struts, and impaired endothelial coverage; these studies and retrospective registry studies also support conservative management in plaque erosion, reinforcing a selective stent-avoidance strategy.^62^^,^^63^ Similarly, pancoronary plaque vulnerability also appears generally lower in erosion, whereas reports of concomitant nonculprit erosions indicate that a conservative strategy may considerably simplify management.^64^ Overall, although identifying erosion may guide stenting and antiplatelet duration decisions, implementation remains constrained by reliance on OCT and absence of validated biomarkers or biomechanical risk tools.

### Toward precision management: biomarkers, biomechanical signatures, and patient susceptibility

A key unmet need in the management of plaque erosion remains the ability to risk-stratify both culprit and nonculprit lesions. In ACS, this involves identifying which patients can be managed safely with antithrombotic therapy alone, whereas in nonculprit segments, it requires detection of erosion-prone plaques. Biomechanical modeling may provide lesion-level risk stratification by distinguishing plaques more likely to destabilize because of adverse mechanical loading, whereas systemic biomarkers reflecting erosion biology, including neutrophil activation and ECM fragmentation products, may offer complementary value.^36^^,^^39^^,^^41^

However, it is important to note that most biomechanical analyses to date are derived retrospectively from reconstructed imaging data sets, which remain primarily as research tools rather than clinical decision-making aids. With advances in automated OCT segmentation and computational processing, biomechanical indices may eventually be derivable in near real time during catheterization and intracoronary imaging workflows, enabling lesion-specific biomechanical profiling.^33^ Such approaches could identify mechanically adverse plaques that do not meet conventional anatomical or physiological high-risk thresholds, including non-flow-limiting or fractional flow reserve–negative lesions. This concept may become increasingly relevant as focal treatment of vulnerable nonobstructive plaques gains interest following the Preventive PCI or Medical Therapy Alone for Vulnerable Atherosclerotic Coronary Plaque (PREVENT) trial, which demonstrated improved outcomes with preventive percutaneous coronary intervention of imaging-defined vulnerable plaques.^65^ In this context, biomechanical profiling may further refine lesion-level selection by identifying which anatomically vulnerable plaques also exhibit adverse mechanical environments and carry heightened destabilization risk. Beyond clinical use, biomechanical markers may serve as research enrichment tools by identifying higher-risk lesions and improving event capture in prospective follow-up studies.

Patient susceptibility to plaque erosion further influences individualized risk stratification. Although sex distribution and age at presentation are similar between erosion and rupture, an age-sex interaction is observed, with erosion occurring more frequently among premenopausal women. Erosion is also associated with fewer traditional metabolic risk factors, whereas smoking is linked to ST-segment elevation myocardial infarction presentations.^1^^,^^8^^,^^66^ Consistent with this, polygenic risk score analyses demonstrated weaker associations between plaque erosion and lipid-associated genetic risk than with rupture, suggesting a distinct host-susceptibility profile.^67^

Together, these observations support a precision framework in which lesion-level biomechanical metrics are integrated with biological and clinical characteristics for refined risk assessment and individualized management. Ultimately, integrated risk models combining anatomical plaque features, biomechanical indices, circulating biomarkers, and patient-level clinical factors may enable more precise risk stratification than any modality alone.

### Emerging therapeutic targets and the future biomechanical agenda

**Figure 5:**
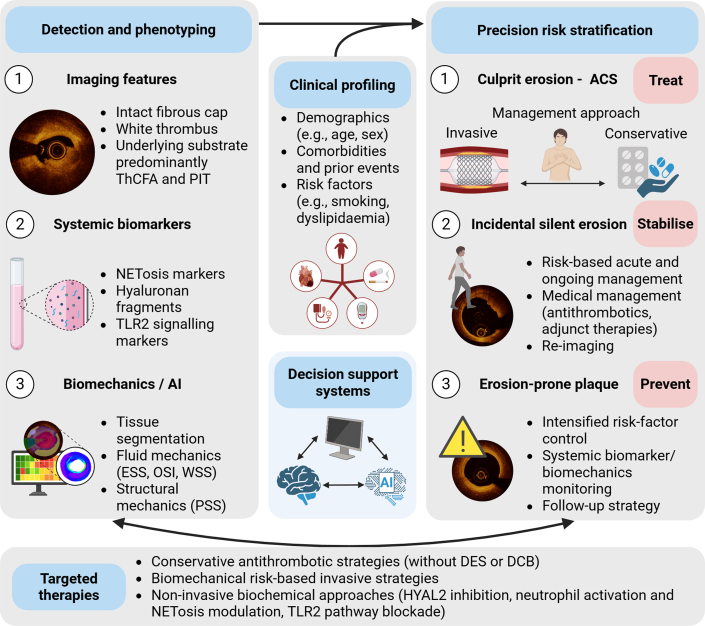
Precision Framework for the Future Recognition and Management of Plaque Erosion Integrated framework for future plaque erosion management. Multimodal detection combines OCT phenotyping, systemic biomarkers, and biomechanics/AI metrics with clinical profiling. Risk stratification distinguishes culprit erosion (ACS), incidental non-culprit (active but silent) erosion requiring stabilization, and erosion-prone plaques (non-active, high-risk phenotype). Management pathways include acute treatment, lesion stabilization, prevention strategies, and emerging targeted therapies such as HYAL2 or NETosis pathway modulation. Created in BioRender. B, I. (2026) https://BioRender.com/o79r1od. ACS = acute coronary syndrome; AI = artificial intelligence; DCB = drug-coated balloon; DES = drug-eluting stent; ESS = endothelial shear stress; HYAL2 = hyaluronidase-2; NET = neutrophil extracellular trap; OSI = oscillatory shear index; PIT = pathologic intimal thickening; PSS = plaque structural stress; ThCFA = thick-cap fibroatheroma; TLR2 = toll-like receptor-2; WSS = wall shear stress.

Insights from erosion biology have revealed several potentially tractable therapeutic pathways (Figure 5). For example, HYAL2-mediated hyaluronan fragmentation amplifies TLR2-driven neutrophil activation, and patients with OCT-defined erosion display increased HYAL2 expression and experimental HYAL2 inhibition reduces monocyte-platelet aggregate formation in vitro, potentially dampening thrombogenic activity in erosion.^39^^,^^68^ NETosis modulation through peptidyl-arginine deaminase-4 or myeloperoxidase inhibition offers another potential avenue.^47^^,^^48^

In parallel, biomechanics offers a complementary opportunity to refine how such therapies are deployed. Lesion-specific mechanical profiling could help identify plaques under destabilizing forces, distinguishing those likely to respond to conservative therapy from those requiring focal intervention. In this way, biomechanical insights may serve to align emerging biological targets with lesion-level risk, supporting more tailored treatment strategies. Although still investigational, this approach highlights the potential for computational modeling to contribute to clinically actionable precision care.

## Conclusions

Plaque erosion is a mechano-biologically distinct cause of ACS in which endothelial denudation, rather than fibrous-cap rupture, initiates thrombosis. Hemodynamic studies implicate adverse shear environments in erosion, particularly low minimum ESS with steep spatial gradients and abrupt plaque topography. Emerging solid mechanics work, including OCT-based FEA or FSI modeling, suggests that structural stress may influence susceptibility to erosion. Together, these insights position erosion as the product of converging mechanical forces, plaque geometry, and a permissive biological milieu that includes endothelial dysfunction and emerging, yet incompletely understood, molecular pathways. Integrating these biomechanical signatures with intracoronary imaging could refine decisions about stent avoidance, guide surveillance of nonculprit segments, and support personalized management. Future research combining biomechanical modeling, molecular biomarkers, and prospective clinical data is essential to validate mechanical erosion predictors and translate biomechanics into precision care.

Perspectives **COMPETENCY IN MEDICAL KNOWLEDGE:** Plaque erosion is a distinct mechanism of acute coronary syndrome in which local biomechanical forces, including endothelial shear stress, plaque geometry, and structural stress, contribute to endothelial injury and thrombosis.

**COMPETENCY IN PATIENT CARE:** Advances in computational biomechanics may allow identification of plaques at risk of future erosion, as well as prognostication of already eroded lesions to facilitate individualized management, including non-interventional approaches in select patients.

**TRANSLATIONAL OUTLOOK:** Prospective studies integrating automated biomechanical modeling with intracoronary imaging, circulating biomarkers, and clinical characteristics are needed to validate biomechanical risk signatures and determine their value for guiding treatment decisions and improving clinical outcomes.

## Funding Support and Author Disclosures

This work was supported by British Heart Foundation Grants PG/18/14/33562, PG/25/12346, RG13/14/30314, RE/24/130011, TA/F/20/210001 (London), Innovate UK Advancing Precision Medicine 10069871, UKRI2943, EPSRC Cambridge Maths in Healthcare (Nr. EP/N014588/1), and Cambridge NIHR Biomedical Research Centres. Drs Roberts and Bennett are founders of Octiocor Ltd, a software imaging company. All other authors have reported that they have no relationships relevant to the contents of this paper to disclose.
