# Supplementary material for: Biomechanical Determinants of Plaque Erosion: Translational Implications and Precision Care
Source: JACC Basic Transl Sci. 2026 Jul 10;11(8):101621. doi: 10.1016/j.jacbts.2026.101621 (PMC13380718; doi:10.1016/j.jacbts.2026.101621)
Supplement: Supplemental Tables 1 and 2 [file mmc1.docx]

**Biomechanical determinants of plaque erosion: translational implications and precision care**

Jason Sangha MD^‡^*, Eirinaios Tsiartas MD, MSc^‡^*, Yuan Huang PhD^‡^, Sophie Gu MD, PhD^‡^, Kevin Mohee MD^‡^, Fan Zhang^¥^, Michael Roberts PhD^¥#^, Martin Bennett MD, PhD^‡#^

^‡^Departments of Medicine and ^¥^Applied Mathematics and Theoretical Physics, University of Cambridge, UK

*Joint first authors

^#^Joint senior authors

**Supplemental material**

**Contents**

**Table S1:** Study eligibility criteria – Page 2

**Table S2:** Search strategy used for records retrieval - Page 3

| **Inclusion criteria** | - Original research (in vivo, ex vivo, and/or computational) articles including abstracts exploring coronary plaque erosion biomechanics (hemodynamic modelling, solid mechanics, or a combination of both). - Use of computational modelling using computational fluid dynamics (CFD), finite element analysis (FEA), or fluid-structure interaction (FSI) models. - Report of quantitative hemodynamic or mechanical parameters, including but not limited to endothelial shear stress (ESS), oscillatory shear index (OSI), plaque structural stress (PSS), radial wall strain (RWS). - Studies involving human participants or human tissue. - Published in English language. - Published at any time since database inception. |
| --- | --- |
| **Exclusion criteria** | - Non-original research articles (e.g., editorials, reviews, letters, case reports). - Studies assessing non-coronary arteries. |

**Table S1:** Study eligibility criteria

| ("plaque erosion"[Title/Abstract] OR "eroded plaque"[Title/Abstract] OR "erosive plaque"[Title/Abstract] OR "intact fibrous cap"[Title/Abstract] OR IFC[Title/Abstract] OR "intimal erosion"[Title/Abstract] OR "endothelial denudation"[Title/Abstract]) |
| --- |
| AND |
| ("biomechanic*"[Title/Abstract] OR "mechanic*"[Title/Abstract] OR "structural mechanics"[Title/Abstract] OR "solid mechanics"[Title/Abstract] OR "hemodynamic*"[Title/Abstract] OR "haemodynamic*"[Title/Abstract] OR "fluid dynamics"[Title/Abstract] OR "finite element analysis"[Title/Abstract] OR "finite-element analysis"[Title/Abstract] OR "FEA"[Title/Abstract] OR "computational fluid dynamics"[Title/Abstract] OR "CFD"[Title/Abstract] OR "fluid structure interaction"[Title/Abstract] OR "fluid-structure interaction"[Title/Abstract] OR "FSI"[Title/Abstract] OR "flow chamber"[Title/Abstract] OR "flow-chamber"[Title/Abstract] OR "simulation"[Title/Abstract] OR "computational model*"[Title/Abstract] OR "multiphysics"[Title/Abstract] OR "wall shear stress"[Title/Abstract] OR "WSS"[Title/Abstract] OR "endothelial shear stress"[Title/Abstract] OR "ESS"[Title/Abstract] OR "shear stress gradient"[Title/Abstract] OR "ESSG"[Title/Abstract] OR "oscillatory shear index"[Title/Abstract] OR "OSI"[Title/Abstract] OR "radial wall stress"[Title/Abstract] OR "circumferential wall stress"[Title/Abstract] OR "wall tension"[Title/Abstract] OR "structural stress"[Title/Abstract] OR "PSS"[Title/Abstract] OR "wall strain"[Title/Abstract] OR "RWS"[Title/Abstract] OR "axial stress"[Title/Abstract] OR "plaque strain"[Title/Abstract] OR "axial wall stress"[Title/Abstract]) |
| AND |
| (coronary[Title/Abstract] OR "coronary artery"[Title/Abstract] OR cardiac[Title/Abstract] OR heart[Title/Abstract] OR myocardial[Title/Abstract]) |

**Table S2:** Search strategy used for records retrieval
